# Ciprofloxacin Release Using Natural Rubber Latex Membranes as Carrier

**DOI:** 10.1155/2014/157952

**Published:** 2014-12-22

**Authors:** Heitor Dias Murbach, Guilherme Jaques Ogawa, Felipe Azevedo Borges, Matheus Carlos Romeiro Miranda, Rute Lopes, Natan Roberto de Barros, Alexandre Vinicius Guedes Mazalli, Rosângela Gonçalves da Silva, José Luiz Ferreira Cinman, Bruno de Camargo Drago, Rondinelli Donizetti Herculano

**Affiliations:** ^1^Biological Sciences Department, Faculty of Language & Sciences, São Paulo State University (UNESP), 2100 Dom Antonio Avenue, 19806-900 Assis, SP, Brazil; ^2^Chemistry Institute, São Paulo State University, 55 Professor Francisco Degni Street, 14800-060 Araraquara, SP, Brazil; ^3^Physics Department, Faculty of Sciences, São Paulo State University, 14-01 Engenheiro Luiz Edmundo Carrijo Coube Avenue, 17033-360 Bauru, SP, Brazil

## Abstract

Natural rubber latex (NRL) from *Hevea brasiliensis* is easily manipulated, low cost, is of can stimulate natural angiogenesis and cellular adhesion, is a biocompatible, material and presents high mechanical resistance. Ciprofloxacin (CIP) is a synthetic antibiotic (fluoroquinolone) used in the treatment of infection at external fixation screws sites and remote infections, and this use is increasingly frequent in medical practice. The aim of this study was to develop a novel sustained delivery system for CIP based on NRL membranes and to study its delivery system behavior. CIP was found to be adsorbed on the NRL membrane, according to results of energy dispersive X-ray spectroscopy. Results show that the membrane can release CIP for up to 59.08% in 312 hours and the mechanism is due to super case II (non-Fickian). The kinetics of the drug release could be fitted with double exponential function X-ray diffraction and Fourier transform infrared (FTIR) spectroscopy shows some interaction by hydrogen bound, which influences its mechanical behavior.

## 1. Introduction

Ciprofloxacin (CIP) is a fluoroquinolone, a synthetic antibiotic of the quinolone drug class [1]. Synthesized in 1981, it is a second-generation antibacterial and recently was pointed out as the most consumed antibacterial agent worldwide and the 5th most commonly prescribed generic antibacterial in the USA [2, 3]. This high level of use, some due to misuse in the sense of unnecessary administration and consumption in irregular dose or with methods neither approved nor supervised by medical professionals, has been blamed for the rapid development of bacterial resistance against this drugs' class [2, 4].

Ciprofloxacin is effective in the eradication of a wide spectrum of Gram-negative and some specific Gram-positive bacteria, including most strains of bacterial pathogens responsible for respiratory, urinary tract, gastrointestinal, and abdominal infections. It is commonly administrated for* P. aeruginosa* osteomyelitis and is widely used as a prophylactic measure in osteomyelitis surgeries [5–7]. The drug shows efficacy and safety in the treatment of adult patients with serious skin and soft tissue infections caused by a variety of bacterial pathogens [8]. The spectrum of activity of the CIP and the location of several injuries which can be treated with this drug open discussion for a new site-specific approach for some infirmities.

Drug encapsulation and dosage reduction as a result of a site-specific approach is perhaps the most convenient way for controlled drug release. The goal of a drug delivery system is to provide the therapeutic dosage at the proper site maintaining the drug concentration during a specific release time. This requires not only a suitable material to hold the drug, and later release it, but also a biocompatible material, with high absorption rate and low rejection. The development of porous carriers had assisted drug delivery systems due to their properties of tunable pore size and well-defined surface properties, allowing a wide manipulation of the carrier in order to control the adsorption and release of drugs in a more reproducible and predictable manner [9]. Herculano et al. showed that the pore density is inversely proportional to the polymerisation temperature of natural rubber latex (NRL) matrix, a strategy that can be used to control drug release [10].

NRL membrane is an important inductor of the healing process of wounds, being used in several medical applications like prosthetics and bone grafts [10–17]. In addition, the treatment of diabetic and phlebopathic ulcers with this membrane leads to a faster healing process due to a vascular growth factor found in the latex and due to a physical blockage of the entrance of new infectious agents in the treated site [10, 12]. To sum up, the NRL membrane has some interesting characteristics such as easy manipulation, low cost, the ability to stimulate natural angiogenesis and cellular adhesion, being a biocompatible material, and the ability to present high mechanical resistance [11].

In this work, a novel release system is proposed based on the encapsulation of CIP in NRL membrane for a sustained and controlled delivery of the drug, possibly being a future application in medicine as surgical bandage for bone and tissue regeneration. Results showed that the NRL membrane can release CIP for up to 312 h, which is relevant for biomedical applications. In addition, the X-ray diffraction (XRD), energy dispersive X-ray spectroscopy (EDS), attenuated total reflection Fourier transform infrared (FTIR-ATR), and mechanical properties are also reported and showed that it is relevant for biomedical applications.

## 2. Materials and Methods

The NRL used in the present study was commercial high-ammonia from BDF Rubber Latex Co. Ltd. (producer and distributor of concentrated rubber latex, Guarantã, Brazil) of about 60% dry rubber content, 4-5% weight of nonrubber constituents such as protein, lipids, carbohydrates, and sugar, and 35% of water [18–20]. After extraction, ammonia was used to keep the latex liquid. The deproteinization of the NRL was performed by centrifugation at 8,000 g. The cream fraction after centrifugation was redispersed to make the desired 60% of dry rubber content latex and then washed twice by centrifugation to reduce the cytotoxic protein content on the solution.

CIP (C_17_H_18_FN_3_O_3_) was purchased from Callithea Pharmaceutics Ltd., Brazil. CIP was incorporated by mixing 3 mL of NRL with 3 mL of drug solution (5 mg/mL). In addition, the drug was found homogeneous (surface and bulk) in the polymer.

These membranes were prepared by pouring the NRL + CIP solution in a stainless steel plate with 5.00 ± 0.05 cm diameter and 200 ± 5.00 *μ*m thickness. Typically the membranes were left for 2 days to fully polymerize at room temperature before use. For the release assay, latex membranes were placed individually in 200 mL of an aqueous solution, from which aliquots were collected during an interval ranging from 10 to 25,000 min. The drug released into the solution was monitored by measuring the UV-VIS spectra with a BEL ENGINEERING SF 200 ADV spectrophotometer, as CIP has a maximum absorption at 275 nm.

In order to describe the kinetics of release from NRL membranes the semiempirical equations (first-order, Higuchi, Hixson-Crowell, Baker-Lonsdale, and Korsmeyer-Peppas) were used. To determine the parameter of the equation the software Sigma Plot 12.5 (from Systat Software) was used. First-order equation occurs due to differences in concentration between the carrier and the media of release (Fickian diffusion) [21], Higuchi equation is applied to slightly soluble one-dimensional matrix that does not swell [22], and Hixson-Crowell equation is used when surfaces dimension diminishes proportionally but the initial geometry keeps constant [21, 23].* In vitro* data were also fitted to Baker-Lonsdale equation, which describes the release from spherical matrices [21, 23]. When the release follows a non-Fickian release, a generic equation as Korsmeyer-Peppas equation can be used, where the value of the release exponent characterizes the release mechanism of drug from matrix [21, 22].

The membranes were characterized by X-ray powder diffraction, using a Siemens D5005 X-ray diffractometer and a graphite crystal as monochromator to select Cu K*α*1 radiation (1.5406 Å), in a step of 0.02° s^−1^. The surface morphology of the NRL membrane was observed using a scanning electron microscope (SEM) model Zeiss EVO 50 (20 KV) and takeoff angle of 35°.

IR spectroscopy of samples was studied by Fourier transform infrared (FTIR) spectrophotometer in the attenuated total reflectance (ATR) mode using a VERTEX 70 (Bruker, Germany) (4000–500 cm^−1^) with resolution of 4 cm^−1^.

Tensile tests were carried out on an EMIC DL2000 fitted with 10 kgf load cell at a speed. The triplicate was pulled at a rate of 500 mm/min (according to ASTM D412) and elongated to failure at room temperature. NRL membranes (44 × 15 × 1.0 mm, length × width × thickness) were prepared with 6 mL of pure NRL or with 2 mg of CIP.

## 3. Results and Discussion

**Figure 1:**
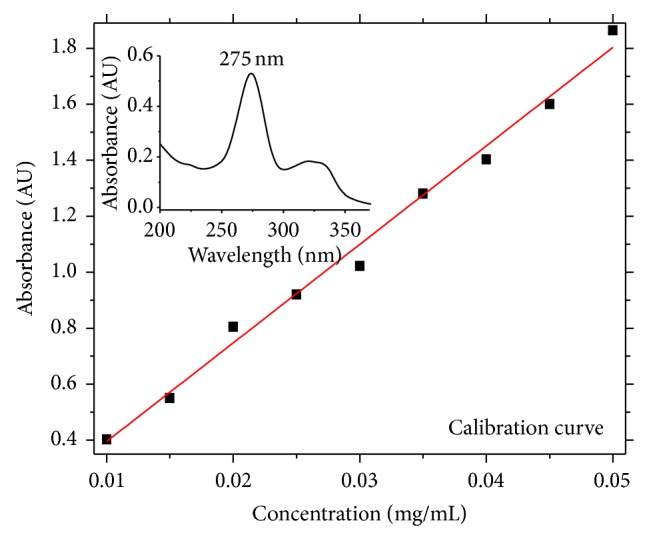
Absorbance intensity as a function of ciprofloxacin concentration in solution.

Pharmaceutical innovation and research are increasingly focusing their attention on the development of delivery systems to enhance desirable therapeutic purposes while minimizing side effects [9, 24]. For the CIP study, the interaction between drug and NRL membrane was evaluated and how it can be incorporated and released from the membrane was also evaluated.  Figure 1 shows the absorbance intensity as a function of CIP concentration in solution. This calibration curve is important to establish a pattern between absorbance and the drug concentration. In this experiment, several drug concentrations from 0.005 to 0.05 mg/mL were used and then the absorbance of the different solutions was measured at 275 nm (spectrophotometer LGS53, BEL Photonics). Using the calibration curve, a sample's concentration can be derived by measuring its absorbance and then finding the corresponding *y*-axis intercept. Note that the graph shows the drug's absorbance range.

**Figure 2:**
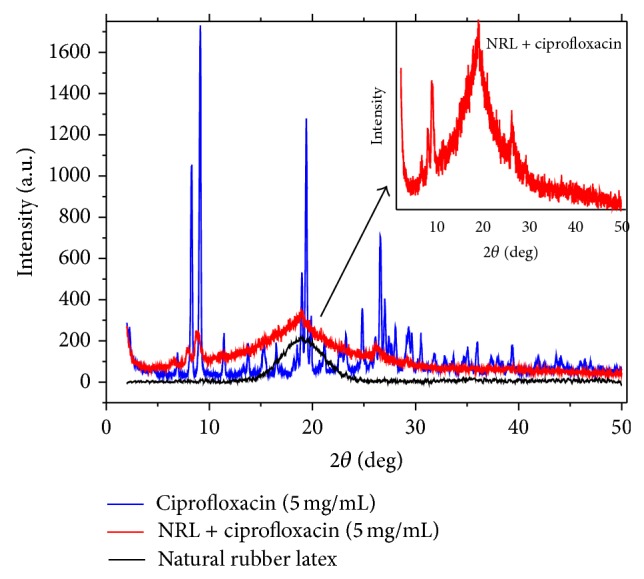
X-ray diffraction pattern of NRL (black line), ciprofloxacin powder (blue line), and NRL membrane prepared with 5 mg/mL of ciprofloxacin (red line).

Figure 2 shows the X-ray diffraction pattern for the NRL membrane, CIP powder, and NRL membrane prepared with 5 mg/mL of CIP, which indicates the amorphous nature of NRL, as expected. In contrast, the drug exhibits an X-ray diffraction pattern of a crystalline material with no amorphous component. Most importantly, its crystallinity was preserved when incorporated into the NRL membrane (the lower-intensity peaks are due to traces of the drug). The changes in the NRL + CIP pattern are caused by the presence of CIP molecules and can be explained by the drug being intercalated in the polymeric structure [9, 25].

**Figure 3:**
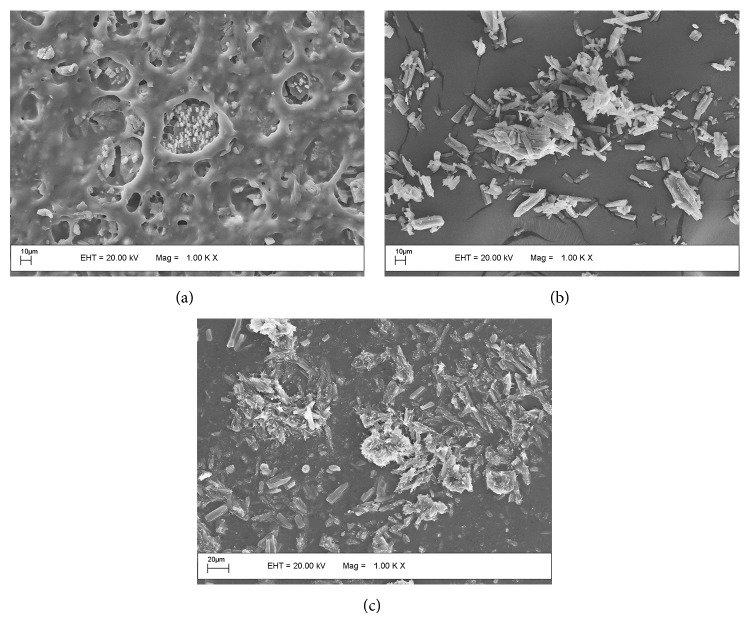
SEM-EDS spectra of (a) NRL membrane, (b) ciprofloxacin powder, and (c) NRL membrane + ciprofloxacin. Note that the drug is present in the NRL matrix.

Figure 3 shows the energy dispersive X-ray spectroscopy (EDS) of a NRL membrane (C_5_H_8_), CIP powder (C_17_H_18_FN_3_O_3_), and NRL + CIP.

**Figure 4:**
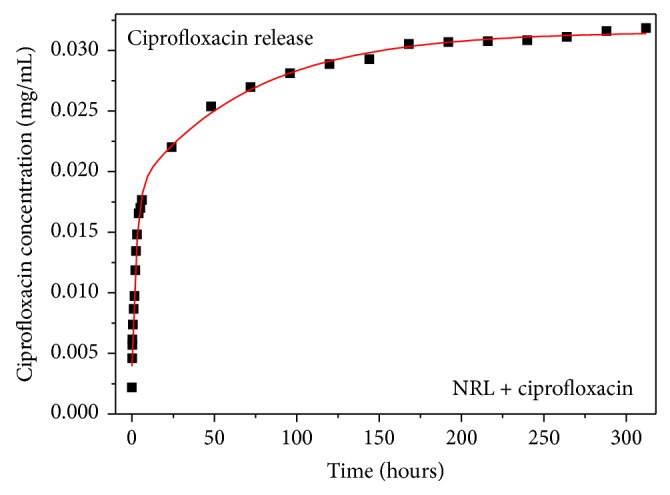
Ciprofloxacin release as a function of time for NRL membrane prepared at room temperature. Notice that the drug concentration reaches a plateau after approximately 170 h.

The release profile of CIP in a NRL matrix in Figure 4 shows saturation at approximately 170 hours. The large bolus of drug released before stable profile rates is called “burst release” (0–25 hours) and it is due to the drug near or adsorbed on the surface of the NRL membrane [24, 26].

The slower release process, also called “stable profile” (25–170 hours), could be associated with the CIP diffusing slowly through the matrix. Thus, the drug is found in the inner portion of the polymeric matrix. The drug release depends mainly on the amount of encapsulated material (as a reservoir).

The experimental data were fitted using a biexponential function *y*(*t*) = *y*_0_ + *A*_1_ *e* ^−*t*/*τ*_1_^ + *A*_2_ *e* ^−*t*/*τ*_2_^, where *y*(*t*) is the amount of CIP in the NRL at a given time, *t*, *y*_0_ is the initial content of the drug, *A*_1_ and *A*_2_ are constants, equal to −0.012 and −0.014, respectively, and the characteristic times are *τ*_1_ = 70.842 hours and *τ*_2_ = 2.548 hours. After integration of these curves until 312 hours, the total amount of drug released by the membrane in 200 mL aqueous solution was 8.86 mg (59.08%).

The parameters from each kinetic model equation are shown in Table 1, and the best fit is Korsmeyer-Peppas equation, due to its high coefficient of determination (*R* ^2^). From the equation *Mt*/*M∞* = *k*∗*t* ^*n*^, where *Mt*/*M∞* (only less than 0.6 should be used) is a fraction of the drug released at time *t*, *k* is the release rate constant (units per time), and *n* is the release exponent.

The *n* characterizes the release mechanism, where *n* < 0.5 corresponds to Fickian diffusion, 0.5 < *n* < 1.0 corresponds to anomalous transport (non-Fickian diffusion), *n* = 1.0 corresponds to relaxation of the polymer fibers (case II transport), and *n* > 1.0 corresponds to super case II.

The *n* obtained indicates that the release mechanism for CIP from NRL is due to super case II. The release from a polymeric matrix is dependent on the solubility of the compound, erosion/degradation, swelling, and relaxation of the carrier [22]. In super case II (non-Fickian) transport mechanism, the velocity of solvent penetration in the carrier is higher than the relaxation and swelling [27] of the polymer; this finding may be due to the natural cross-linking in the NRL and the low swelling and degradation due to its hydrophobicity [28]. Similarly, Verma et al. [27] demonstrated that the super case II transport also happens to some formulations of chitosan to release CIP.

**Figure 5:**
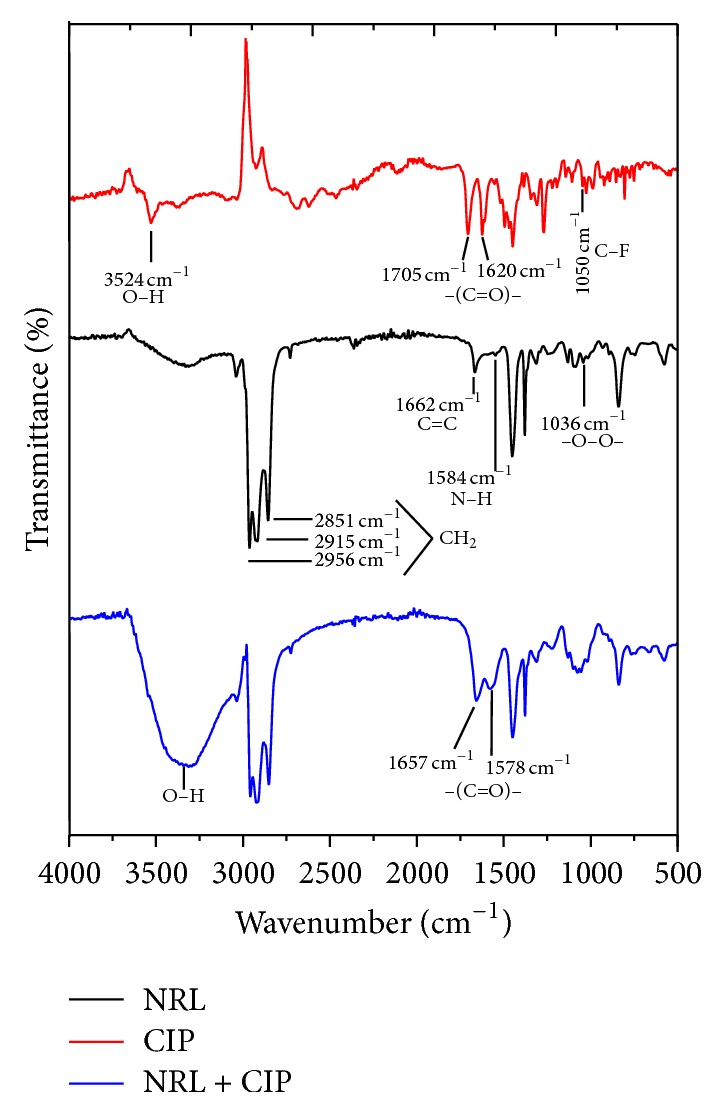
FTIR-ATR spectra of ciprofloxacin, NRL, and NRL loaded with ciprofloxacin.

Figure 5 shows the FTIR-ATR spectra of CIP, NRL, and NRL loaded with CIP. In all spectra, absorption band from 3500 to 3450 cm^−1^ is due to the stretching vibrations of the hydroxyl group or the intermolecular hydrogen bonding.

From FTIR spectra of CIP (Figure 5), it was found that the peak at 3524 cm^−1^ is attributed to the stretching vibrations of O–H of the carboxylic group. At 1705 and 1620 cm^−1^ the peaks are due to the stretching vibrations of the carbonyl group of carboxylic acid and ketone, respectively. The 1444 cm^−1^ peak is attributed to C–H bending or C–O, the 1271 cm^−1^ is due to C–C–C of ketone, and the 1045 cm^−1^ is due to C–F [27, 29–31].

The NRL FTIR spectra showed the isoprene absorption peaks at 2956 and 2915 cm^−1^ due to CH_2_ asymmetric stretching, at 2851 cm^−1^ it is due to CH_2_ symmetric stretching, at 1662 cm^−1^ it is due to C=C stretching, at 1371 cm^−1^ it is due to CH_3_ asymmetric deformation, and at 840 cm^−1^ it is due to =CH out-of-plane bending [32]. Furthermore, functional groups of phospholipids and proteins have also been found at 1584, 1217, and 1036 cm^−1^ that are related to N–H bending, C–O, and –O–O–, respectively [28]. The FTIR spectra of the NRL incorporated with CIP showed interaction. The overlapped band in the region of 3700–3200 cm^−1^ and the slight shift of the bands attributed to the carbonyl group indicate some interaction, most likely by hydrogen bonding, which were also observed on chitosan [29].

**Figure 6:**
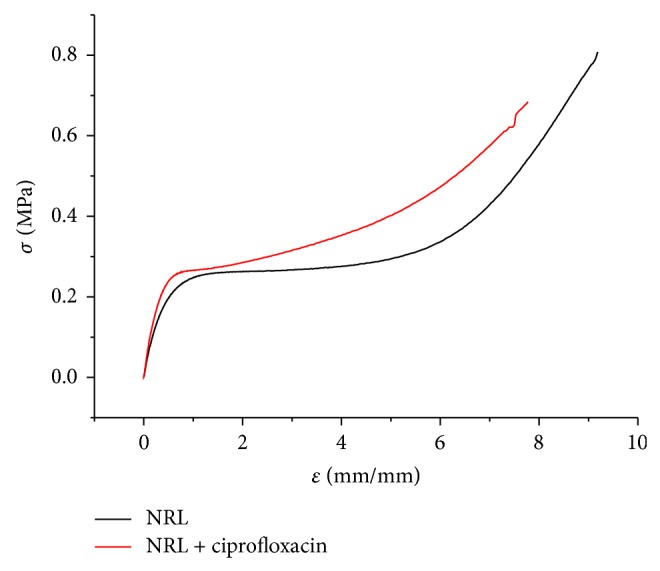
Representative stress-strain curves NRL and ciprofloxacin loaded NRL.

The addition of CIP to the NRL membrane had influence on the mechanical behavior of NRL (Figure 6). The new material became stiffer and brittle, with a smaller plastic deformation (less ductile).  Table 2 shows that the addition of CIP reduced 1.2 times the elongation at break and reduced the tensile strength.

The increase of hydrogen bonding with the incorporation of CIP indicates interaction of the drug with rubber macromolecules, leading to higher interfacial interactions, acting as reinforcement or creating cross-linking [33] which resulted in loss of elasticity [34].

In this work, the method proposed by Langer and Folkman was used [35], that is, to mix the protein with the polymer (latex) in a colloidal state, in order to create a membrane that works as a delivery system.

Already Löbler et al. [36] developed a device based on polyhydroxyalkanoates (PHA) for implantation of a glaucoma drainage system. In this study, PHA based on hydroxybutyric acid was tested in terms of its potential suitability to manufacture mechanically stable tube components of drug delivery drainage systems and in terms of biocompatibility.

Herculano et al. [10] proposed a drug release system based on NRL for the sustained and controlled delivery of metronidazole (MET). They concluded that the release time of MET in* in vitro* tests was very promising for the kinetics of release.

Wang et al. [37] prepared uniform-sized chitosan microspheres by membrane emulsification technique. Uniform chitosan microspheres were further used as a carrier of a protein drug bovine serum albumin (BSA). They observed that BSA loading efficiency was highest when pH value was 8.09, and it decreased with an increase of the cross-linking degree.

The controlled release of proteins is of interest for medical applications, since the dose can be adjusted according to the application envisaged. The results indicate that, with very simple changes in preparation of NRL membrane, it is possible to control CIP release up to 13 days, according to Wang et al. [37], Woo et al. [38], Herculano et al. [39, 40], Malcolm et al. [41], and Langer and Folkman [35] results.

## 4. Conclusion

We have prepared NRL membranes containing CIP as a model system for tissue and bone regeneration. The method of preparation is reproducible and the NRL membrane is very stable. The results indicate that NRL could be a future candidate to be used as a drug delivery membrane. From the results obtained with FTIR and mechanical behavior assay, hydrogen bonding interactions between CIP and NRL membrane can be observed. Nevertheless, the NRL membrane was able to release 59.08% of the drug in 13 days and could be fitted by a biexponential equation which can help to predict the release of the drug. The Korsmeyer-Peppas kinetic model of release indicates that the mechanism of release is due to super case II transport (non-Fickian diffusion). In addition, the X-ray spectroscopy technique shows that the drug did not interact chemically with the membrane. Likewise, the crystal structure of CIP was essentially maintained, which shows the encapsulation of the drug within the amorphous membrane. The SEM-EDS and the release assay indicated that CIP was present both inside and on the surface of the polymeric matrix, thus making it a promising material for drug release in* in vivo* applications. The possibility of a new treatment method for osteomyelitis and skin wound with a surgical bandage made of NRL and CIP is promising. The use of lower drug doses and the control of drug delivery and site-specific release may improve the healing process and the quality of life of the patient and, in addition, can reduce the indiscriminate use of antibiotics. Further research is required to fulfill these predictions, such as* in vitro* studies focusing on the release rate and time and porosity required to achieve therapeutic effect and* in vivo* models to study the efficiency of the surgical bandage theory. Other carriers can be tested, as alginate-chitosan [42], carrageenan [43], and pectin [44], which might also be promising candidates; however the characteristics and availability of NRL in Brazil make it an attractive material for further investigation.

## Figures and Tables

**Table 1:** Kinetic parameters of equations for mechanism of release 5 mg/mL.

	*R* ^2^	*k* (units per hour)	*n*
Baker-Lonsdale equation	0.88	1.10 × 10^−8^	Not applied
Korsmeyer-Peppas equation	1.00	8.00 × 10^−4^	1.15
Hixson-Crowell equation	0.99	6.08 × 10^−6^	Not applied
Higuchi equation	0.89	2.57 × 10^−2^	Not applied
First-order equation	0.99	1.83 × 10^−5^	Not applied

**Table 2:** Mechanical properties.

	Breaking force	Tensile strength	Elongation at break	Young's modulus
	(N)	(MPa)	(%)	(MPa)
NRL + CIP	9.23 ± 0.57	0.58 ± 0.085	750.00 ± 22.61	0.86
NRL	10.95 ± 0.85	0.76 ± 0.019	911.60 ± 12.60	0.68
